# Salivary protein 7 of the brown planthopper functions as an effector for mediating tricin metabolism in rice plants

**DOI:** 10.1038/s41598-022-07106-6

**Published:** 2022-02-25

**Authors:** Gu Gong, Long-Yu Yuan, Yi-Feng Li, Hang-Xiang Xiao, Yan-Fang Li, Yang Zhang, Wei-Jian Wu, Zhen-Fei Zhang

**Affiliations:** 1 Guangdong Provincial Key Laboratory of High Technology for Plant Protection, Plant Protection Research Institute, Guangdong Academy of Agricultural Sciences, Guangzhou, 510640 Guangdong People’s Republic of China; 2 College of Plant Protection, South China of Agricultural University, Guangzhou, 510640 Guangdong People’s Republic of China

**Keywords:** RNAi, Gene expression analysis

## Abstract

The brown planthopper (BPH), *Nilaparvata lugens*, is an important pest that affects rice (*Oryza sativa*) production in Asia. The flavone tricin (5,7,4′-trihydroxy-3′,5′-dimethoxy flavone) is a valuable secondary metabolite commonly found in rice plants that can defend rice plants against infestation by BPH. BPH damage can reduce the metabolic level of tricin in rice. Our preliminary transcriptome research results showed that BPH salivary protein 7 (NlSP7), is highly responsive to tricin stimuli. However, the function of NlSP7 in mediating the interaction between the rice plant and the BPH is unknown. In this study, we cloned the *NlSP7* gene in *N*. *lugens* and found that its mRNA level was greater in the presence of high tricin content than low tricin content, regardless of whether the BPHs were fed a rice plant diet or an artificial diet containing 100 mg/L tricin. Knocking down *NlSP7* resulted in BPH individuals spending more time in the non-penetration and pathway phase, and less time feeding on the phloem of rice plants. These changes decreased BPH food intake, feeding behavior, and fitness, as well as the tricin content of the rice plants. These findings demonstrate that the salivary protein 7 of BPH functions as an effector for tricin metabolism in rice.

## Introduction

Herbivorous insects obtain nutrients from host plants. As sessile organisms, many plants have evolved chemical defense mechanisms to resist these herbivorous insects^1^. For example, plants can initiate defense mechanisms when they detect insect oral secretions or signals from damaged plant cells. Many secondary metabolites produced by plants result from this co-evolutionary struggle between herbivores and plants^2^. Recent evidence indicates that saliva-secreted proteins can mediate host plants’ defense responses^3^. The saliva of herbivorous insects thus plays a key role in plant–insect interactions and in mediating the defenses of host plants^4^.

The brown planthopper (BPH), or *Nilaparvata lugens* Stål (Hemiptera: Delphacidae), is a major rice (*Oryza sativa* L.) pest in Asia^5^. Rice plants have evolved a variety of physical and chemical defenses against the BPH. Tricin (5,7,4′-trihydroxy-3′,5′-dimethoxy flavone) is a valuable secondary metabolite that is widely distributed in the stems, leaves, and hulls of many gramineous plants, including rice^6^. Previous studies show that tricin has an ecological function against the BPH by eliciting anti-feeding or anti-oviposition behavior^7,8^. In rice plants, the flavone tricin 5-O-glucoside is a probing stimulant for the white-backed planthopper (*Sogatella furcifera* Horvath). Other study has shown that tricin of BPH-resistant rice variety IR36 significantly reduces mortality rates of the rice and reduces honeydew weights of BPH, tricin also inhibits their oviposition and feeding behaviors^9^. Electrical penetration graph (EPG) data show that tricin significantly inhibits in vivo and in vitro movement of the BPH stylus^6^.

The BPH is a typical phloem sap-sucking insect. The first salivary protein identified in BPHs was β-glucosidase^10^. Rice plants treated with β-glucosidase react with increased salicylic acid, ethylene, and hydrogen peroxide levels and decreased jasmonic acid content^11^. Other salivary proteins, such as salivary sheath protein (*NlShp*), salivary endo-b-1,4-glucanase (*NlEG1*), mucin-Like Protein (*NlMul*), and anther mucin-Like Protein (*NlMLP*), are shown to play roles in the BPH’s salivary sheath formation and feeding^12–15^. NlEG1 degrades cellulose in the plant cell wall to allow the stylet of a BPH to reach the phloem^14^. NlMul-deficient BPHs exhibit disordered development and sometimes death^16^. In plants, NlMLP induces cell death, expression of defense-related genes, and callose deposition^15^. However, less is known about the role of salivary effectors of BPHs in mediating the chemical defenses of rice.

In this study, we aim to fill the knowledge gap by exploring the function of the NlSP7 salivary protein in mediating the interaction between rice plants and BPHs. Specifically, we assess whether NlSP7 acts as an effector for mediating tricin metabolism in rice plants. Our previous study has shown that the relationship between rice plants and BPHs is an ideal model to reveal plants’ defense mechanisms at the molecular level and the effects of secondary metabolites on BPHs^17^. The results of this study can help in developing novel BPH-resistant rice cultivars.

## Results

### Characterization of *NlSP7*

**Figure 1:**
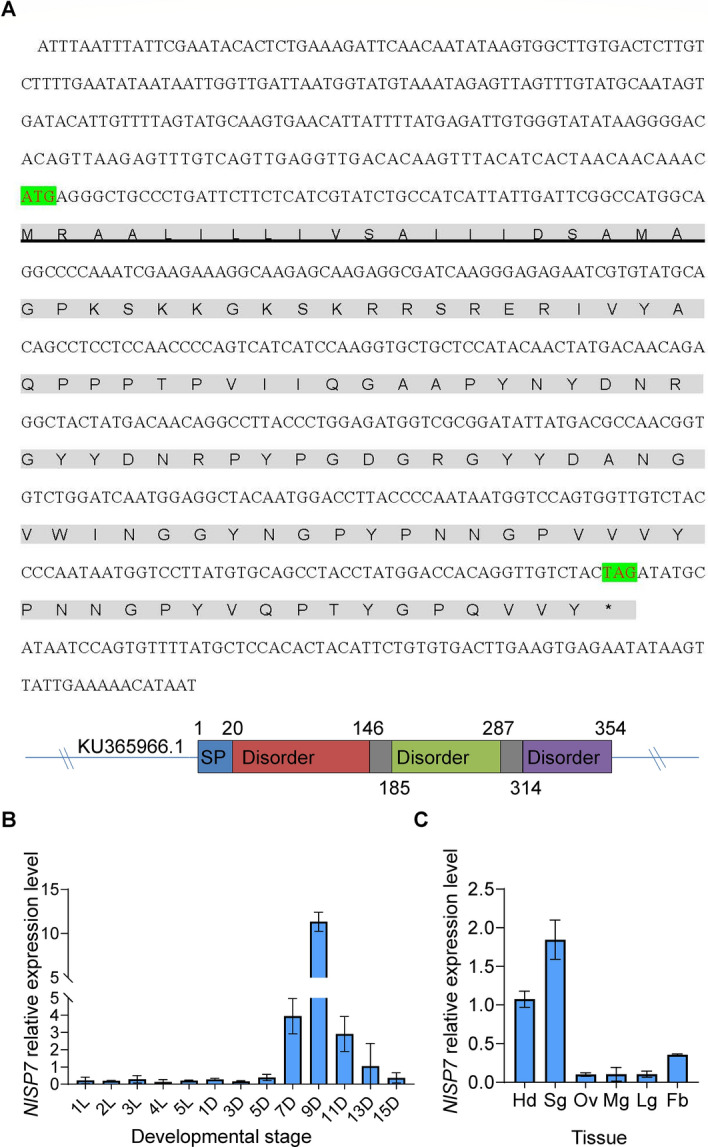
Characterization of *NlSP7*. (**A**) CDS sequence of *NlSP7*. The solid underline indicates the signal peptide as predicted by SignalP 5.0. The asterisk (*) indicates the stop codon. The different short-repeat regions are indicated by different colors. (**B**,**C**) The bottom panels show the mean transcript levels ± SD (n = 30) of *NlSP7* in whole bodies at various developmental stages (**B**) and in different tissues (**C**). RNA was extracted from nymphs at 1–5 days and female brachypterous brown planthoppers reared on Rathu Heenati rice at 1, 3, 5, 7, 9, 11, 13, and 15 days. Hd, head; Sg, salivary gland; Ov, ovary; Mg, midgut; Lg, leg; Fb, fat body was extracted from BPH adults.

Using the proteomics database established for BPH by our laboratory^18^, we obtained a homologous fragment (312 base pairs) based on alignments with known insect transcriptome sequences. The open reading frame of the sequence was amplified via PCR with gene-specific primers. The complete sequence (678 base pairs) of the cDNA was obtained by RACE, and it comprised a 242-bp 5′ untranslated region, 355-base pair open reading frame, and 81-base pair 3′ untranslated region (Fig. 1A). The predicted protein comprised 117 amino acid residues with a theoretical molecular mass of 12.88 kDa. We designated this sequence as *NlSP7* (GenBank accession no. KU365966.1) and *NlSP7* sequences showed significant similarities with no similar protein of other insect species.

To investigate the spatio-temporal expression pattern of *NlSP7* in 13 different developmental stages (1st, 2nd, 3rd, 4th and 5th instar nymphs, 1st, 3rd, 5th, 7th, 9th, 11th, 13th, 15th days old adults) and various parts of the adults (head, salivary glands, ovaries, midgut, leg, and fat body) of BPH. RT-qPCR showed that *NlSP7* transcripts were expressed in all nymph and adult stages (Fig. 1B). The significantly higher expression in adults suggests that *NlSP7* plays its most important role in the adult stage. Tissue-specific expression analysis in adults showed that the *NlSP7* transcript levels were slightly higher in the salivary glands, followed by the head and fat body, with lowest expression in the ovaries, midgut, and fat body (Fig. 1C).

### *NlSP7* expression activated by tricin in different rice varieties

**Figure 2:**
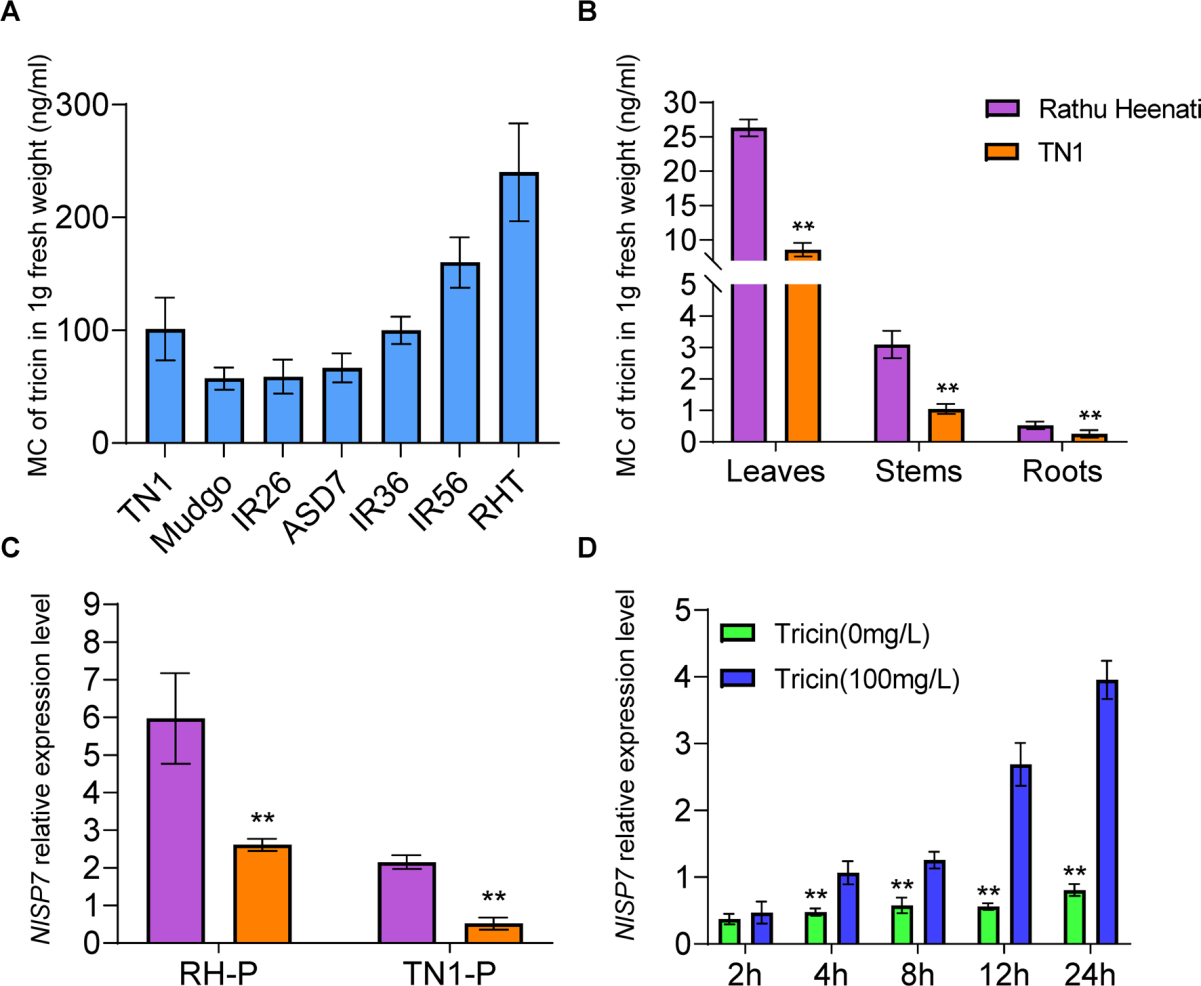
Relationship between *NlSP7* and tricin. (**A**) Tricin mass concentration (MC) (ng/ml) analysis results at the three-leaf stage of seven rice varieties: Taichung Native 1 (TN1), Mudgo, IR26, ASD7, IR36, IR56, and Rathu Heenati (RH) carrying different genes resistant to brown planthoppers. (**B**) Tricin mass concentration of tricin in the root, stem and leaf parts of RH and TN1 rice varieties. One g each of fresh root, stem, and leaf was ground, and the content of tricin in each part was measured using liquid chromatography-mass spectrometry. Data are represented as the mean ± SD from three biological replicates. (**C**) q-PCR was used to determine the expression level of TN1-P and RH-P fed two different varieties of rice plants. (**D**) Artificial diets wih tricin and without tricin were fed to brown planthoppers, then q-PCR was used to detect the expression level of *NlSP7* at 2, 4, 8, 12, and 24 h. The mean amounts of three biological replicates (mean ± SD, n = 10) are shown, ** P < 0.01. (Student’s *t*-test).

Tricin concentrations in the different rice varieties and organs were different (Fig. 2). The tricin identification results for the TN1, Mudgo, IR26, ASD7, IR36, IR56, and RH varieties were shown in Fig. 2A. The tricin concentrations in the leaves were significantly higher than those in the stems and roots in both the resistant RH and susceptible TN1 varieties. There were no significant differences in the tricin concentrations between the stems and roots. The tricin concentration in the leaves was 28.1 ± 2.6 ng/ml in the RH plant, which was significantly higher than that in the leaves of the TN1 plant (*P* = 0.002). The tricin concentration in the RH roots differed significantly from that in the TN1 roots (*P* = 0.004) (Fig. 2B).

First, the optimal concentration of tricin had been chosed from two different tricin concentrations (50 mg/L and 100 mg/L) (Supplementary Fig. S4). The resistence levels differed significantly for the tricin-resistant RH population of BPHs and tricin-sensitive TN1 population of BPHs. The mean resistence levels were significantly lower for the tricin-sensitive population (*P* < 0.001) than the tricin-resistant population when feeding on the RH rice variety (control in this study, severity score = 9.0) (Fig. 2C). The *NlSP7* gene mRNA expression level was higher in the tricin-resistant BPHs than in the tricin-sensitive population (Fig. 2C). These results suggest that *NlSP7* may have a role in BPH feeding and virulence. BPH nymphs fed an artificial diet containing tricin had higher *NlSP7* transcript levels from 4 h, compared with those fed a diet without tricin (*P* < 0.01) (Fig. 2D). Thus, the *NlSP7* mRNA levels were upregulated after ingesting an artificial diet containing tricin.

### Fitness of BPH

To elucidate the role of *NlSP7* in BPHs, we synthesized dsRNA from *NlSP7* and injected it into 1-day-old female adults to mediate RNAi (Supplementary Fig. S1). This treatment had strong silencing effects, reducing the *NlSP7* transcript level significantly (~ 86%) in the first day after treatment, compared with the levels in the control group, in those injected with ds*GFP* RNA (*P* < 0.01), and in those injected with ds*NlSP7* (*P* = 0.001 after injection with ds*GFP* or ds*NlSP7* at 2 days, *P* < 0.001 after injection with ds*GFP* or ds*NlSP7* at 3 days).

**Figure 3:**
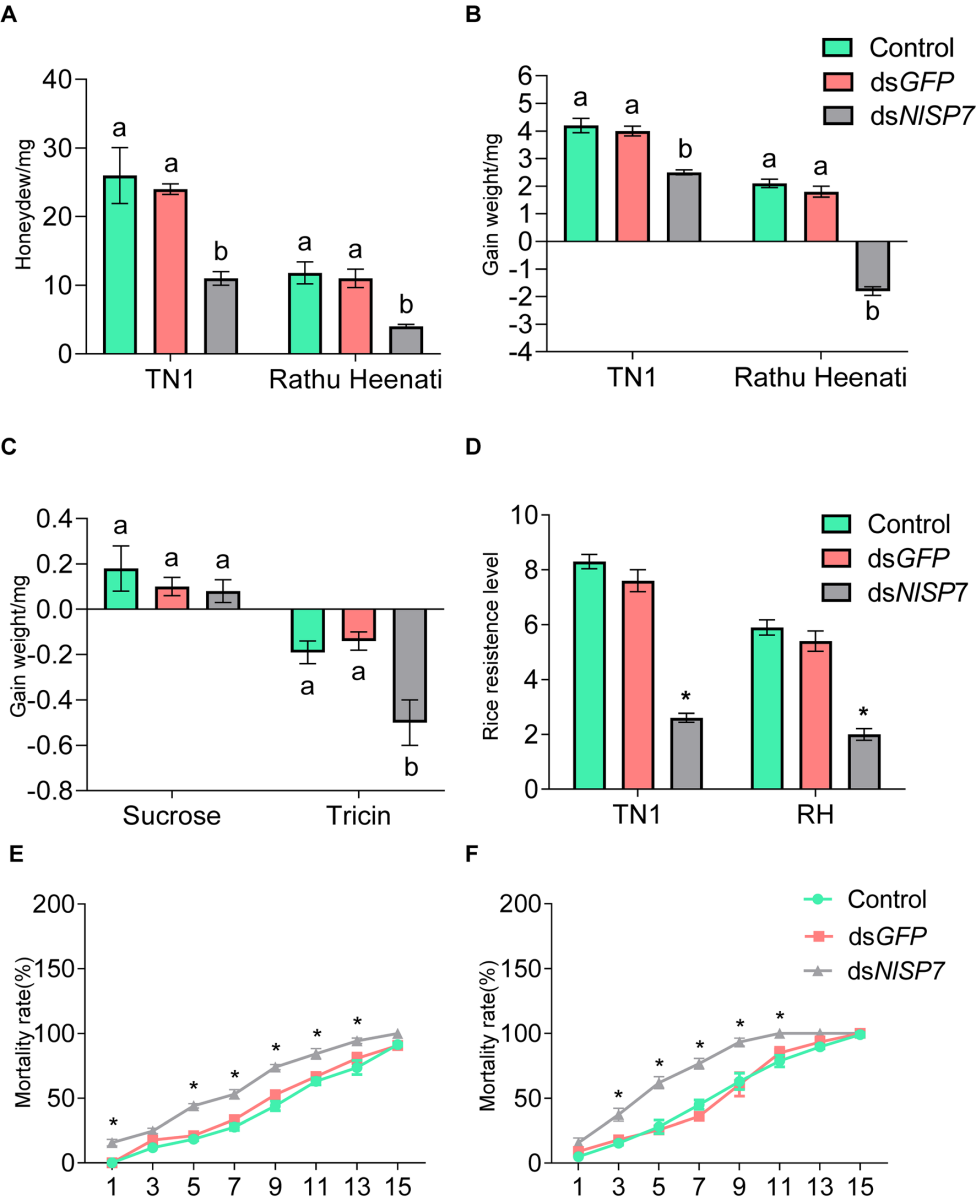
RNA interference (RNAi) effects on feeding of brown planthoppers (BPHs) exposed to three different treatments (control, injected with ds*GFP*, injected with ds*NlSP7*) and resistance levels of different rice varieties. (**A**) On Taichung Native 1 (TN1) and Rathu Heenati (RH) rice varieties, the mean honeydew amounts excreted at 24 h by a female adult BPH that received three treatments. The experiment was repeated with 20 replicates. (**B**) Weight gain of BPHs fed TN1 and RH rice varieties after injection with different dsRNA treatments. (**C**) Mean weight of BPHs fed LDS-sucrose and LDS-tricin (mean ± SD, n = 20). Letters indicate significant differences (*P* < 0.01, Duncan’s multiple range test). (**D**) Rice resistance levels of BPHs fed TN1 and RH rice varieties. The experiment was repeated three times. Data are represented as the mean ± SD (*P* < 0.01, Duncan’s multiple range test). (**E**) mortality rate of BPHs fed RH rice, repeated 10 times, respectively. (**F**) mortality rate of BPHs fed an artificial diets with 100 mg/l tricin, repeated 10 times. Asterisks (*) indicate significant difference (*P* < 0.05, Student’s *t*-test).

BPHs subjected to the *NlSP7* RNAi treatment also exhibited less feeding activity, as indicated by significantly lower excretions of honeydew, compared with the two control groups (*P* < 0.001 for control and ds*NlSP7*; *P* = 0.003 for ds*GFP* and ds*NlSP7*), as shown in Fig. 3A. The *NlSP7*-RNAi BPHs also had lower weight gain values, regardless of whether they were fed RH rice plants (*P* < 0.001 for control and ds*NlSP7*, *P* = 0.001 for ds*GFP* and ds*NlSP7*) or TN1 rice plant (Fig. 3B), and smaller weight gain values (*P* = 0.006 for the control and ds*NlSP7*, *P* = 0.003 for ds*GFP* and ds*NlSP7*), as shown in Fig. 3C. Furthermore, silencing *NlSP7* reduced the virulence of BPH (Fig. 3D)^15^. Compared with the two control groups, BPHs injected with ds*NlSP7* had significantly lower survival rates from 3 to 11 days after microinjection (*P* = 0.003, *P* = 0.003, respectively), and most died on RH rice by 9 days after microinjection (Fig. 3E). Similar results were fed an artificial diet containing tricin (100 mg/L) (Fig. 3F). The BPHs injected with ds*NlSP7* fed on TN1 rice, the mortality rate was no difference between the BPH injected with ds*NlSP7* and the control group, which were feeding on artificial diet without tricin. (Supplementary Fig. S2 and S3).These results indicate that silencing the *NlSP7* gene significantly reduces feeding and performance of BPHs on rice plants.

### Impairment of BPH feeding

**Figure 4:**
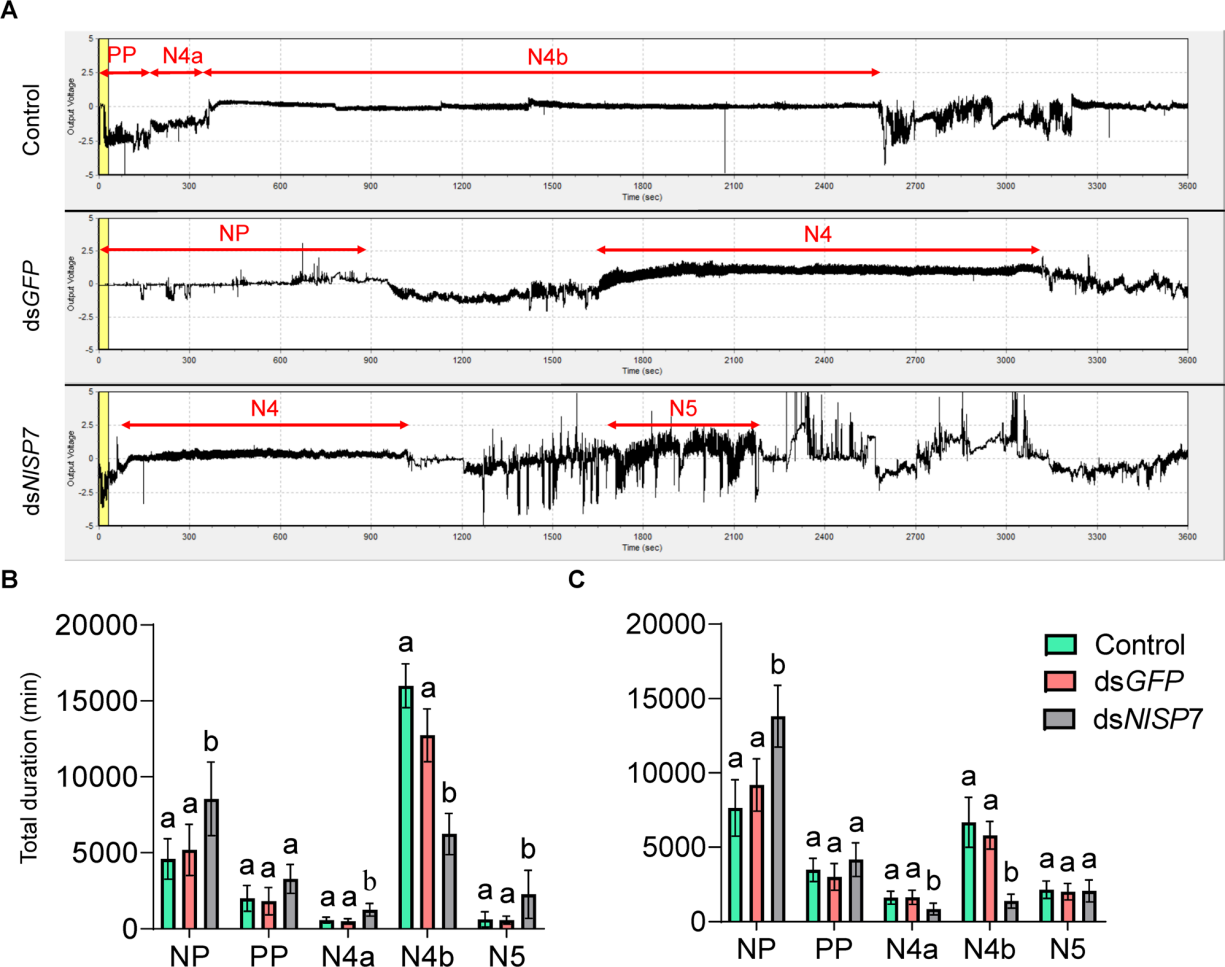
Reduced feeding on Taichung Native 1 (TN1) and Rathu Heenati (RH) rice varieties among three groups of brown planthoppers (the order is control, injected with ds*GFP,* injected with ds*NlSP7*). (**A**) Overall typical view of electrical penetration graph waveforms generated by feeding behavior of the three groups of brown planthoppers. (**B**,**C**) one-day-old brachypterous female adults in the three treatment groups. NP, nonpenetration; PP, pathway phase (N1 + N2 + N3), including penetration initiation (N1), salivation and stylet movement (N2), and extracellular activity near the phloem (N3); N4a, intracellular activity in the phloem region; N4b, phloem sap ingestion; N5, xylem phase. Duration of wavelength when each insect feeds on TN1 or RH rice. Electrical penetration graphs were recorded for 6 h per insect. Different letters indicate significant differences among the three treatment groups. Testing of each group was repeated 15 times (*P* < 0.01, Duncan’s multiple range test).

**Figure 5:**
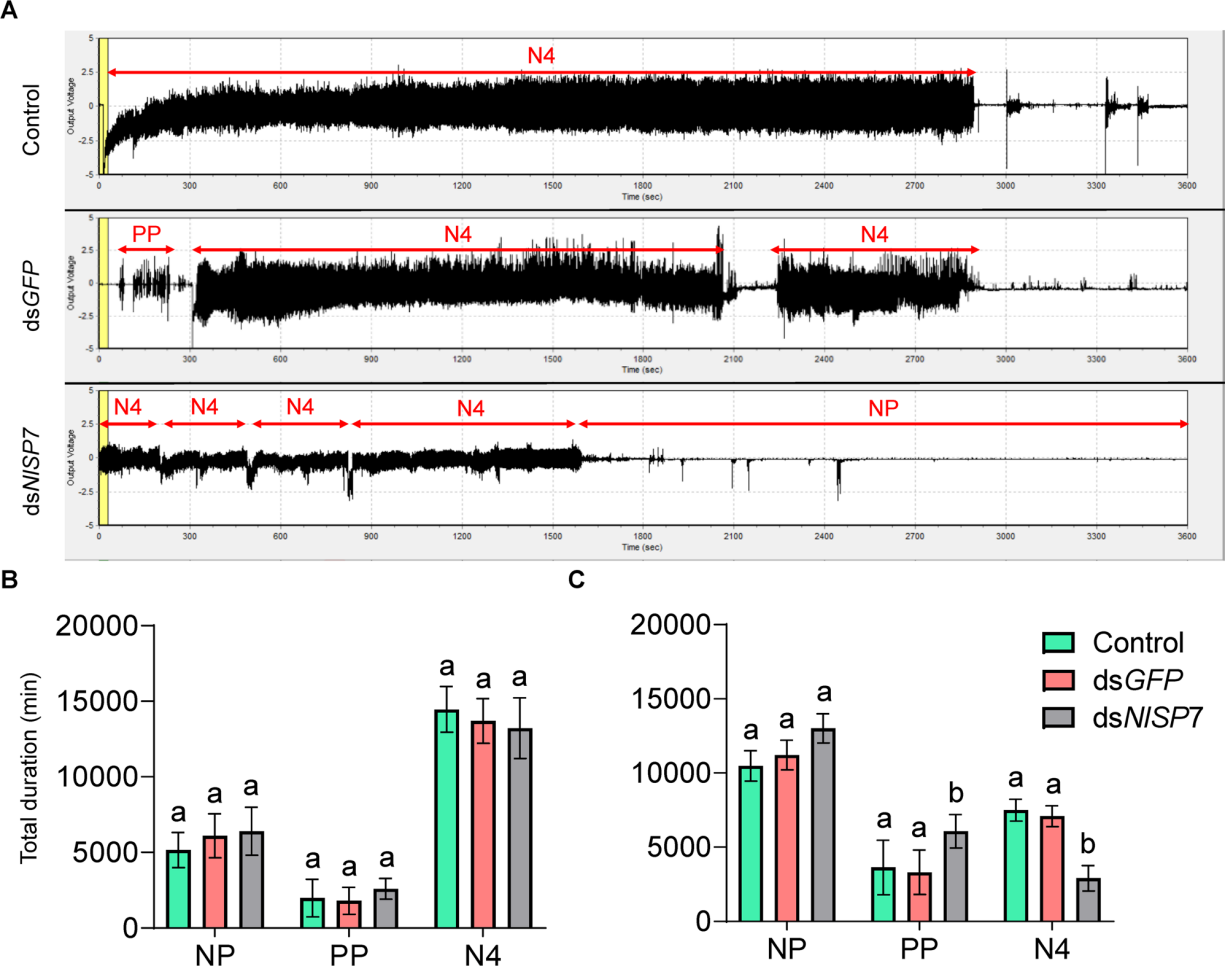
Brown planthoppers in three groups (the order is control, injected with ds*GFP*, injected with ds*NlSP7*) exhibit reduced feeding on two artificial diets (0 mg/l tricin and 100 mg/l tricin). (**A**) Overall typical view of electrical penetration graph waveforms generated by the feeding behavior of brown planthoppers on artificial diets. (**B**,**C**) data collected from 1-day-old brachypterous female adults with different treatments. NP, nonpenetration; PP, pathway phase (N1 + N2 + N3), including penetration initiation (N1), salivation and stylet movement (N2), and extracellular activity near the phloem (N3); N4, sucrose or tricin solution ingestion. B the duration of each wavelength when feeding on artificial diets without tricin (0 mg/l) and C artificial diets with tricin (100 mg/l) is shown. Electrical penetration graph data were recorded for 6 h per insect. Different letters indicate significant differences among the three treatment groups. Testing of each group was repeated 15 times (*P* < 0.01, Duncan’s multiple range test).

To determine the effect of *NlSP7* on BPH feeding, we used the EPG technique to profile the feeding behavior of piercing–sucking insects^19^. Five main feeding phases can be distinguished by EPG: (1) the non-penetration phase, (2) the pathway phase (including penetration, salivation, stylet movement, and extracellular activity near the phloem), (3) the intracellular phase of activity in the phloem, (4) the phloem sap ingestion phase, and (5) the xylem phase. The top of Fig. 4A shows the representative EPG traces obtained from BPHs, showing the different phases. As shown in Fig. 4B, after *NlSP7* knockdown in female adults, the EPG waveforms obtained over 6 h on TN1 rice plants showed that the duration of intracellular activity in the phloem exceeded those of the ds*GFP* and control groups (*P* = 0.001), and the duration of the N4b did the duration decrease, other waveforms have increased in varying degrees, the non-penetration (NP) the intracellular activity in the phloem region(N4a) and the xylem phase(N5) increased, the pathway phase (PP) was unchanged for dsNlSP7-treated insects (*P* < 0.001, *P* = 0.011, *P* = 0.001, *P* = 0.007, respectively). On RH rice plants, the duration of the non-penetration phase increased significantly (*P* < 0.001), whereas the durations of the intracellular activity in the phloem and phloem sap ingestion phases decreased significantly over the 6-h period (*P* = 0.017, *P* < 0.001, respectively), as shown in Fig. 4C. These findings indicate that BPH individuals spent less time feeding on rice plants with high tricin levels after *NlSP7* knockdown. The EPG distinguishes three main feeding stages in artificial diets, the non-penetration (NP), the pathway phase (PP) and the artificial diets ingestion (N4) (Fig. 5A). After *NlSP7* knockdown in female adults, there are no obvious change in the EPG waveforms obtained over 6 h on artificial diets without tricin (Fig. 5B), and the EPG waveforms obtained over 6 h on artificial diets with tricin showed that the duration of pathway phase activity in the phloem exceeded those of the ds*GFP* and control groups (*P* = 0.001), the N4 duration reduced (Fig. 5C). These results of artificial diets indicate that BPH individuals spent less time feeding on artificial diets with tricin levels after *NlSP7* knockdown.

**Figure 6:**
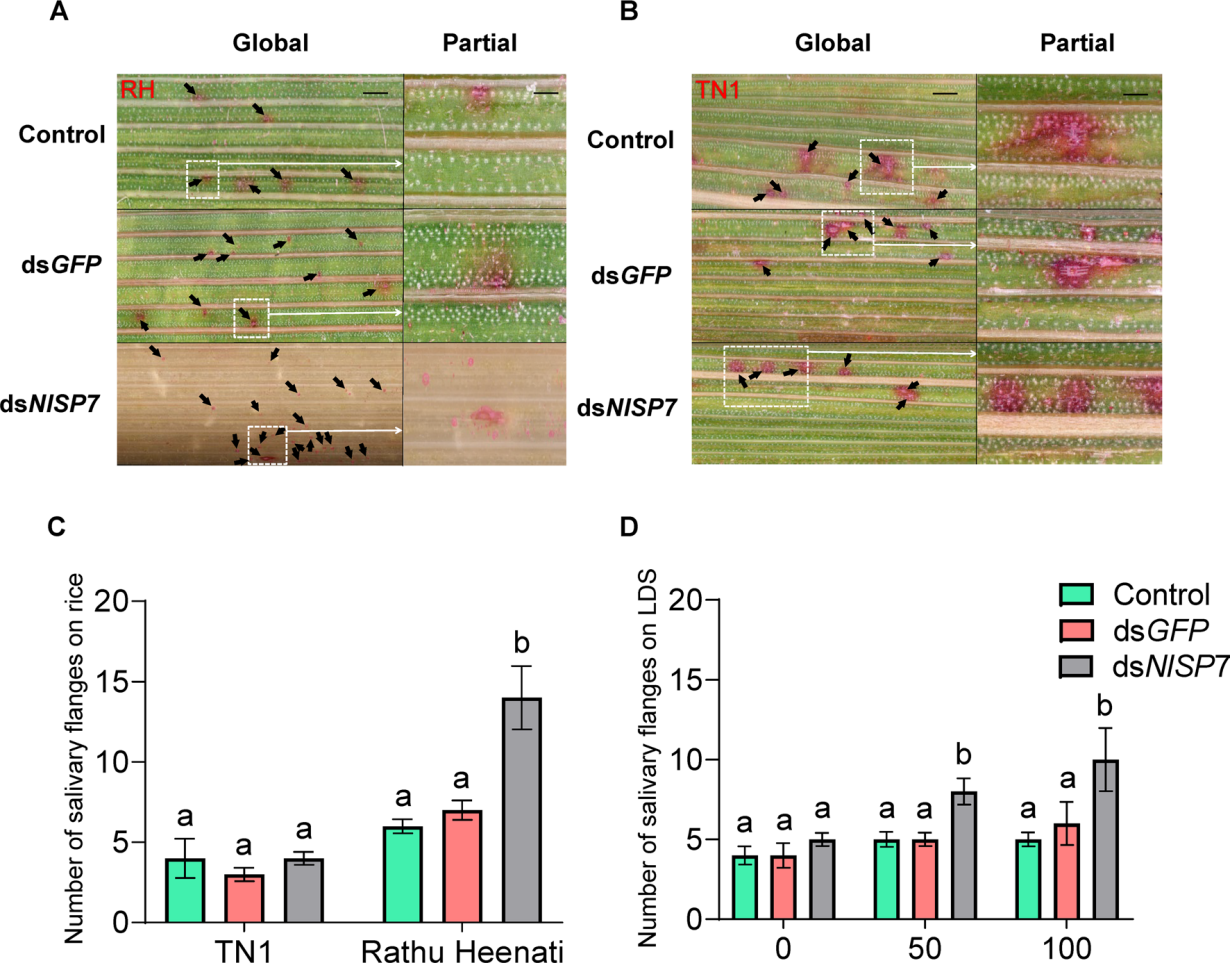
Number of salivary flanges created by brown planthoppers fed rice or an artificial diet, across different treatment groups (control, injected with ds*GFP*, injected with ds*NlSP7*). (**A**,**B**) images of salivary flanges produced on Rathu Heenati (RH) and Taichung Native 1 (TN1) rice varieties. The global of graphs, bars = 200 μm. The partial of graphs, bars = 50 μm. (**C**) Salivary flanges created by brown planthoppers fed different rice varieties. (**D**) Salivary flanges from brown planthoppers fed artificial diets with different levels of tricin. Bars with different letters are significantly different among different tricin levels. Data are represented as means ± SD, with 30 replications (*P* < 0.01, Duncan’s multiple range test).

Furthermore, BPHs fed an artificial diet high in tricin produced significantly more salivary flanges than those in the control group and those fed an artificial diet containing low tricin levels (*P* < 0.001) (Fig. 6D). In addition, the number of salivary flanges differed significantly between the two rice plant varieties. BPHs produced 250% more salivary flanges on RH plants than on TN1 plants after *NlSP7* knockdown (Fig. 6A,B). Moreover, comparing salivary flanges among the control group, the numbers of salivary flanges were sparser per unit area and deeper on TN1 plants than on RH plants (Fig. 6C).

### Tricin metabolism mediated by NlSP7

**Figure 7:**
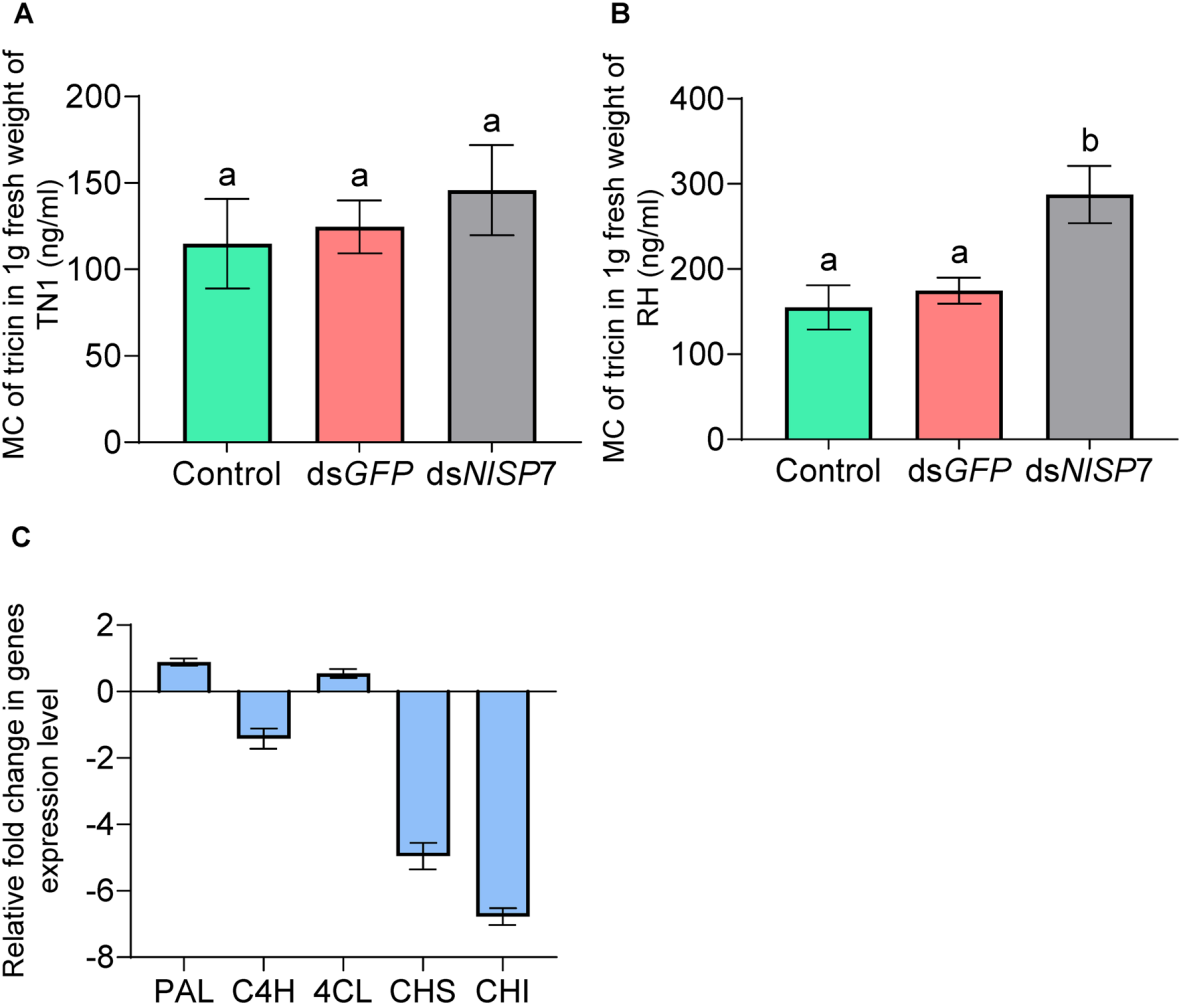
The effect of brown planthoppers in three groups (control, injected with ds*GFP*, injected with ds*NlSP7*) on the tricin content and tricin metabolic pathway genes of different rice varieties. (**A**) Taichung Native 1 (TN1) and (**B**) Rathu Heenati (RH) rice varieties fed on by brown planthoppers. After 3 days, the content of tricin was determined by liquid chromatography-mass spectrometry, A same letters above the bars indicate no significant differences between three treatment groups, and B different letters above the bars indicate significant differences between BPHs injected with ds*NlSP7* and two control groups (control and ds*GFP*). Data are represented as the mean ± SD from three biological replicates (*P* < 0.01, Duncan’s multiple range test). (**C**) The q-PCR used to determine the expression level of tricin metabolic pathway genes (PAL, C4H, 4CL, CHS, CHI) in rice after being fed on by brown planthoppers. The experiment was repeated three times.

To determine whether the salivary protein NlSP7 influences the production of tricin in rice plants, we investigated tricin levels in rice plants infested by BPH adults, where the ability to produce *NlSP7* was silenced or not silenced. The results showed that RH rice plants fed on by control and ds*GFP* groups BPH had decreased tricin levels than BPH group which injected by ds*NLSP7* and RH rice plants fed on by BPH adults had decreased tricin levels compared to RH rice plants not fed on by BPHs, but there is no significant change between the TN1 treatment group and the control group (Fig. 7A,B). After *NlSP7* knockdown, the tricin level in RH rice plants increased by 86.36% (*P* = 0.005) but remained unchanged in TN1 rice plants. Tricin is a secondary metabolite of the phenylalanine pathway in plants, and the phenylalanine pathway starts from phenylalanine. The amino group is removed under the action of phenylalanine ammonia lyase (*PAL*) to form cinnamic acid. Cinnamic acid adds hydroxyl to form p-coumarin acid under the action of cinnamic acid-4-hydroxylase (*C4H*), and then forms p-coumarin acid COA under the catalysis of 4-coumarin-CoA ligase (*4CL*). Chalcone synthase (*CHS*) catalyzes the formation of chalcone, the isomerization of chalcone to flavanone, which is catalyzed by chalcone isomerase (*CHI*)^20^. To further characterize the physiological properties associated with the blocked synthesis of flavonoids induced by *NlSP7*, we examined the expression levels of PAL, C4H, 4CL, CHS, CHI for the synthesis of flavonoids in RH rice plants fed by BPH for 5 days (Fig. 7C). Our results indicated that the level of tricin induced by *NlSP7* shared similarities with the secondary metabolite synthetase induced by the flavonoid pathway marker genes CHS and CHI. Thus, *NlSP7* may play important roles in defense-related signal transduction^21^.

## Discussion

Saliva is a complex mixture of biomolecules, and it plays crucial roles in how sap-sucking insects feed on plants^12–16^. Our experiments demonstrated that the NlSP7 secretory protein in the salivary gland of BPHs is injected into rice plants during feeding. The protein encoded by *NlSP7* contains three unknown domains; it is unique to BPHs and exhibits typical tandem amino acid duplication, which is consistent with previous analyses^22^. In our previous studies, we collected watery and gelling saliva from BPHs to characterize the salivary proteome^18^. BPH saliva also includes a large effector repertoire involved in its interaction with rice plants, according to previously reported evaluations of the salivary proteome^23^ and watery salivary proteome^24^. However, for the first time, our results showed that *NlSP7* is involved in the interaction between BPH and defensive secondary metabolites in rice plants.

Tricin is an active secondary metabolic with great potential for combating BPHs by eliciting anti-feeding or anti-oviposition behavior, according to previous studies^7,8^ and confirmed by our results. In the present study, we prepared a tricin-resistant BPH population that could feed on both RH rice (high tricin content) and TN1 rice (low tricin content). Plant-derived compounds play key roles in insect–plant interactions, and insects can adapt to changes in the levels of these compounds^25^. *NlSP7* may act as an herbivore effector that allows BPH to overcome the defensive effect of tricin in rice plants, as indicated by our results showing that *NlSP7* was more highly expressed in the tricin-resistant population than in the tricin-sensitive population. *NlSP7* knockdown significantly reduced transcript levels and honeydew quantity by 57.69% and 63.64% in BPHs feeding on TN1 and RH rice varieties, respectively, and weight gain decreased on both the RH rice diet and artificial diet containing 100 mg/L of tricin. The mortality rate reduced by 5% among BPHs fed the RH rice variety (*P* < 0.01) and by 15% among BPHs fed the artificial diet containing 100 mg/L tricin (*P* < 0.01). These results are similar to those reported in studies of two other saliva genes, *NlShp* and *NlEG1*. Knocking down *NlShp* by RNA interference inhibited salivary sheath formation, silencing *NlEG1* decreases the capacity of BPH to reach the phloem. They have similar results, which will cause brown planthoppers to have difficulty feeding^13,14^.

Larvae release saliva into plant cells through wound sites created when they feed on the leaves of plants^23,26^. After *NlSP7* gene knockdown in tricin-resistant BPHs, the duration of feeding on rice plants decreased significantly. BPHs fed the high-tricin RH rice variety spent more time in the non-penetration and pathway phases and less time feeding on phloem, thereby decreasing their food intake, weight gain, and survival rate. However, silencing *NlSP7* did not affect the early ability of BPH to feed on an artificial diet containing 100 mg/L tricin. These results suggest that *NlSP7* facilitates phloem access via cell wall penetration by interacting with tricin.

Our EPG results showed that the duration of intracellular activity in the phloem and phloem sap ingestion phases decreased significantly after *NlSP7* knockdown. The durations of the non-penetration and pathway phases increased significantly among BPHs that were fed either rice plant variety or an artificial tricin-rich diet. The number of salivary flanges in the two different plant types and under various tricin levels indicated that ingestion of tricin induced significant increases in salivary flanges. The expression of *NlSP7* in the saliva could have affected tricin-induced feeding of BPHs. Similar to other salivary proteins, the expression of *NlEG1* in the fat body might be related to the detoxification of plant defense chemicals, as reported previously for some plant cell wall-degrading enzymes in insects^27^.

The elicitation of tricin production in rice plants as a defense reaction by *NlSP7* shares common features with the immune responses of well-known effectors and pathogen-associated molecular processes. It also may be recognized by plant pattern recognition receptors, which trigger plant defensive responses. The rice plant’s defense response to BPHs involves secondary metabolites, which is a common response triggered by insect feeding^4,28,29^. Our results suggest that the increased duration of the non-penetration and pathway phases induced by *NlSP7* silencing were dependent on the tricin content. We consider that plants might respond to *NlSP7* via a conserved upstream component of plant signaling pathways. The expression of *NlSP7* decreased the expression levels of the flavonoid pathway marker genes *CHS* and *CHI*. *CHS* is a chitinase gene associated with flavonoid-dependent defenses, and it is induced by elicitors^30^. The isomerization of naringenin chalcone to synthesize naringenin flavanone, which is catalyzed by *CHI*^31^. These findings suggest that the defense responses triggered by *NlSP7* are associated with the flavonoid signaling pathway. Inhibition of the flavonoid signaling pathway promotes the synthesis of lignin, which leads to increased thickening of plant cell walls^32^. A previous study showed that BPHs feeding on the RH rice variety could lead to thickening of the plant cell wall^33^. Increasing the thickness of the cell wall on the sieve plates might occlude the sieve tubes enough to directly inhibit continuous feeding by BPHs^28^. It is thus reasonable to suggest that phloem plugging also might cause dsNlSP7 to perform poorly in BPHs. Further research is required to address this question.

The results obtained in this study indicate that *NlSP7* may be an effector involved in the interactions between rice plants and BPHs. The expression level of *NlSP7* was higher in the salivary gland of the tricin-resistant BPH population. After *NlSP7* knockdown, the amount of honeydew produced by dsRNA-treated BPHs feeding on TN1 and RH rice varieties decreased by 57.69% and 63.64%, respectively, and the virulence scores decreased by 56.16% and 75.84%, respectively. *NlSP7* also plays an important role in BPH feeding, as we found that the durations of sucking and phloem feeding decreased by 47.42% and 79.36%, respectively, when BPHs fed on the RH rice variety. The protein encoded by *NlSP7* can form a complex with interacting partners when secreted into rice plants.

We found that *NlSP7* could influence the tricin content in rice. *NlSP7* may activate rice defense responses by affecting the expression of flavonoid biosynthesis genes in plants. Further studies are needed to identify the interacting partner of *NlSP7* in rice plant varieties and to determine the role of the complex. The novel molecular interaction identified in this study may provide new insights on the interaction between BPH and defensive secondary metabolites in rice. Further investigation is needed to assess the usefulness of this interaction in rice agriculture.

## Materials and methods

### Insects and plant materials

Rice variety Taichung Native 1 (TN1) is a BPH-susceptible rice variety with a low concentration of tricin. Rathu Heenati (RH) has a high tricin content^6^, all plant materials to comply with relevant institutional, national, and international guidelines and legislation. Both varieties of seeds were obtained from the experimental nursery of the Plant Protection Research Institute, Guangdong Agricultural Academy of Science, China. All seeds for experiments were germinated on filter paper, placed in a Petri dish, and transferred to a pot (7 cm in diameter) containing multi-purpose compost. The plants were then maintained in a growth chamber at 27 ± 0.5 °C with a relative humidity of 70 ± 5% and a 12-h light/dark cycle. Plants aged 30 days were used in the experiments.

The *N. lugens* populations used for collecting secreted saliva in this study originated from Guangdong Agricultural Academy of Science, Plant Protection Research Institute, in Guangzhou, Guangdong Province, China. The tricin-resistant population was fully reared for at least 35 generations and maintained on RH rice plants. The insects were reared at 27 ± 0.5 °C at a relative humidity of 70 ± 5%. Host plants were replaced weekly. Only 1-day-old adult BPH females were selected for use in experiments.

### Standards and reagents

Tricin, high-performance liquid-chromatography grade ethyl acetate (> 99.8%), and high-performance liquid-chromatography grade methanol (> 99.9%) were purchased from Merck; MS grade methanol (> 99.9%) from Thermo Fisher Scientific (Waltham, MA, USA); and analytical reagent methanol from Donghong Chemical Factory (Guangzhou, China). The reagents used for molecular experiments were purchased from TransGen Biotech and Genstar (Guangzhou, China). 2,4,5–7-tetra bromo fluorescein disodium salt was purchased from Shanghai Yuanye Bio-Technology Co., Ltd. (China).

### Reverse transcription PCR and sequence analysis of *NlSP7*

Newly molted 1-day-old brachypterous female adult BPHs were anesthetized with carbon dioxide for 15 s and dissected in phosphate-buffered saline (pH 7.4). The head, salivary gland, midgut, ovary, leg, and fat body were carefully dissected from 100 individuals under a stereomicroscope (Leica S8AP0, Wetzlar, Germany) before placing the tissues directly in 200 μL of TRIzol for total RNA extraction. After preparing 30 replicate BPHs, total RNA was isolated using a TRIzol Total RNA Isolation Kit (Takara, Dalian, China) according to the manufacturer’s instructions. The total RNA concentration was quantified, and 1,000 ng of RNA was used for reverse transcription in 20 μL of reaction volume using a TransScript® II One-Step gDNA Removal and cDNA Synthesis SuperMix kit (TransGen Biotech, Beijing, China) according to the manufacturer’s instructions. Next, 5′ and 3′ rapid amplifications of cDNA ends (RACE) was conducted to amplify *NlSP7* cDNA using the 5′-full RACE kit and 3′-full RACE core set (TaKaRa, Kyoto, Japan) according to the manufacturer's instructions. Supplemental Table S1 shows the specific primers for *NlSP7*.

PCR products were cloned into carriers using a pEASY®-Blunt Zero Cloning Kit (TransGen Biotech,Beijing, China) and sequenced by IGEBio Technology Ltd. (Guangzhou, China). The full-length cDNA sequence of *NlSP7* was assembled from the sequencing results and verified by PCR using the primers shown in Table S1. The sequence with a signal peptide was submitted to TMHMM Server version 2.0 to predict transmembrane domains (http://www.cbs.dtu.dk/services/TMHMM/). SignalP 5.0 was used to predict the presence of signal peptides (http://www.cbs.dtu.dk/services/SignalP/). Identified N. lugens sequences were verified using BLASTX to search for similar sequence in National Center for Biotechnology Information (NCBI) protein database.

### Real-time quantitative PCR (RT-qPCR)

Real-time quantitative PCR analyses were conducted with reverse transcription cDNA. The RT-qPCR analyses were using CFX96 Real-Time PCR Detection System (Bio-Rad,USA) using 2 × RealStar Green Fast Mixture. The pcr program uses a two-step method: initial denaturation at 95 °C for 2 min followed by 40 cycles of 95 °C for 15 s and 60 °C for 15 s. *Nlactin* (GenBank:EU179848) were used as the reference gene to normalize gene transcript levels (Table S1). The relative gene transcript levels and interference efficiency were calculated using the 2^-△△CT^ method.

### RNAi

Double-stranded RNA (dsRNA) synthesis was conducted based on the cloned *NlSP7* sequences. A nucleotide sequence (around 300 bp long) specific to the target gene was cloned into the pEASY® Blunt Zero vector (TransGen Biotech, Guangzhou, China) and submitted for sequencing to IGE Biotechnology Ltd. (Beijing, China). *Aequorea victoria* green fluorescent protein (GFP) was used as a control. PCR products were used as templates for dsRNA synthesis using a MEGAscript T7 transcription kit (Thermo Fisher, Waltham, MA, USA). Supplemental Table S1 shows the specific primers used to generate these DNA templates.

Newly molted 1-day-old brachypterous female adults were first anesthetized with carbon dioxide for 15 s and then injected with approximately 100 ng of dsRNA using a Nanoject II Auto-Nanoliter Injector (Drummond Scientific, Broomall, PA, USA). After injection, the treated BPHs were reared on RH rice at 27 ± 0.5 °C at a relative humidity of 70 ± 5% for 3 days. The injected BPHs were used in the experiments.

### Electrical penetration graph (EPG) recording

EPG was performed using a Giga-8 DC EPG amplifier (Wageningen Agricultural University, Wageningen, The Netherlands). At 1 h before the EPG experiments, the 1-day-old brachypterous female adult BPHs were provided with water on filter paper, then anesthetized with CO_2_ for 20 s^6^. Newly molted 1-day-old brachypterous female adults were selected from the insect cages and subjected to one of three different treatments: control, injection with double-stranded GFP (ds*GFP*), or injection with ds*NlSP7*), with 15 replicates for each treatment. BPHs were fixed using a negative pressure device.

One end of a 3-cm length of gold wire (diameter = 12.5 μm) was connected to an amplifier through the EPG probe. The other end was connected to the dorsum of the BPH with conductive silver glue (Wageningen Agricultural University). A copper electrode (length = 10 cm, diameter = 2 mm) was inserted into the soil in the RH or TN1 planting substrate to establish the other part of the electrical circuit. After a starvation period of 30 min, the BPH was placed on a rice plant. All EPG experiments were recorded for 6 h under continuous light in a climate-controlled room. The 6 h of continuous EPG data starting from the beginning of feeding were analyzed using EPG Stylet + software (Wageningen Agricultural University, 2012).

The artificial diet system comprised a transparent and open cylindrical container (diameter = 6 cm, height = 5 cm) with a double layer of Parafilm PM-996 (Bemis, Oshkosh, WI, USA) covering one end of the container^6^. The experimental treatments tested two concentrations: 100 mg/L tricin and no tricin. One end of a copper wire was embedded in the solution, and the other end was wrapped around the electrode. The insect electrode and experimental control conditions were used to obtain the reference EPG data for a plant.

### BPH bioassays for honeydew and weight gain

Honeydew is used to measure BPH feeding activity. Honeydew was collected as described by Pathak et al.^34^. One-day-old brachypterous female adults were injected with *NlSP7* or *GFP* dsRNA or not injected (control) and placed on RH rice plants. After 3 days, the active BPHs were placed into Parafilm bags (6 cm × 5 cm) fixed on the stem of an RH or TN1 rice plant and maintained for 24 h in a climate-controlled environment at 27 ± 0.5 °C with a relative humidity of 70 ± 5% and a 12-h light/dark photoperiod. Twenty replicates were prepared.

To assess the effects of *NlSP7* knockdown on feeding, BPHs subjected to three treatments (control, injected with ds*GFP*, or injected with ds*NlSP7*) were allowed to feed on different rice plant varieties or on an artificial diet. A single rice plant was placed in a round plastic tube (length = 10 cm, diameter = 2 cm) with a hole to facilitate breathing. The treated BPHs were weighed and placed in the tubes. After feeding for 24 h, the BPHs were removed and weighed again. Changes in weight were determined for BPHs fed an artificial diet and those subjected to the three treatments. Data were collected in the same manner as for the rice-fed BPHs. Twenty replicates were prepared for each treatment.

### Salivary flange measurements

A single rice plant was placed in a round plastic tube (length = 10 cm, diameter = 2 cm). One-day-old female adult BPHs subjected to one of three treatments (control, injected with ds*GFP*, or injected with ds*NlSP7*) were placed in plastic tubes sealed with sponges. The BPHs were maintained in a climate-controlled environment at 27 ± 0.5 °C at a relative humidity of 70 ± 5% and a 12-h light/dark photoperiod. After 24 h, BPHs and the rice stalks were removed. The rice stalks were soaked in Eosin Y for 12 h before observing the salivary flanges on the stalks under a stereoscope. The salivary flanges from BPHs fed an artificial diet were observed directly under a microscope. Thirty replicates were prepared for each treatment.

### Liquid chromatography-mass spectrometry

Liquid chromatography and mass spectrometry analyses were conducted using a DGU-20A3 (Shimadzu, Kyoto, Japan) quaternary pump equipped with an autosampler. A Supelco Discovery C18 XDB-C18 (Agilent, Santa Clara, CA, USA) column (4.6 × 50 mm, 1.8 μm) was used at ambient temperature with a sample injection volume of 10 μL. The elution gradient comprised a binary solvent system with H_2_O (solvent A) and mass spectrometry-grade methanol (solvent B) at a constant flow rate of 800 μL/min. Mass spectrometry and tandem mass spectrometry experiments were performed using a hybrid triple quadrupole/linear ion trap API4000 Q-Trap liquid chromatography mass spectrometer (Applied Biosystems, Carlsbad, CA, USA)^6^.

### Statistical analysis

All data in this article are expressed as the mean ± standard deviation. The content of tricin in different parts of different rice varieties and the expression levels of *NlSP7* in different BPH populations (e.g., those fed on TN1 or RH rice varieties) were determined using Student’s *t*-test. Duncan’s multiple range test was used to analyze the expression level of BPH after feeding on artificial diets with tricin for 2, 4, 8, 12 and 24 h; the bioassay of BPH after RNA interference; and the changes in tricin content after feeding on different rice varieties. All statistical tests were performed using SPSS 17.0 software (SPSS Inc., Chicago, IL, USA).

### Ethical approval

This article does not contain any studies with human participants or animals performed by any of the authors.

## Supplementary Information

Supplementary Information.
