# Supplementary material for: Salivary protein 7 of the brown planthopper functions as an effector for mediating tricin metabolism in rice plants
Source: Sci Rep. 2022 Feb 25;12:3205. doi: 10.1038/s41598-022-07106-6 (PMC8881502; doi:10.1038/s41598-022-07106-6)
Supplement: Supplementary file 1 — Supplementary Information. [file 41598_2022_7106_MOESM1_ESM.docx]

**Supporting information**

**Salivary protein NlSP7 of the brown planthopper functions as an effector for mediating tricin metabolism in rice plants**

Gu Gong ^1, 2, #^, Long-Yu Yuan ^1, #^, Yi-Feng Li ^1^, Hang-Xiang Xiao ^1^, Yan-Fang Li ^1^, Yang Zhang ^1^, Wei-Jian Wu ^2^, Zhen-Fei Zhang ^1, *^

^1^ Guangdong Provincial Key Laboratory of High Technology for Plant Protection, Plant Protection Research Institute, Guangdong Academy of Agricultural Sciences, Guangzhou 510640, P R China

^2^ College of Plant Protection, South China of Agricultural University

* Correspondence: Zhen-fei Zhang, Guangdong Provincial Key Laboratory of High Technology for Plant Protection/Plant Protection Research Institute, Guangdong Academy of Agricultural Science, Guangzhou 510640, China.

E-mail address: zhangzhenfei@gdaas.cn

^#^ These authors contributed equally to this work.

Declarations of interest: none.

***Content***

**Supplementary Figure S1.** Interference efficiency of NLSP7.

**Supplementary Figure S2.** Mean mortality rates of BPH female adults on TN1 rice.

The experiment was repeated three times with 100 BPHs per replicate.

**Supplementary Figure S3.** Mean mortality rates of BPH female adults on artificial diets.

The experiment was repeated three times with 100 BPHs per replicate.

**Supplementary Figure S4.** Salivary flanges of BPH female adults on artificial diets.

The experiment was repeated three times with 100 BPHs per replicate.

**Supplementary Table S1.** Primers used for qRT-PCR and PCR.


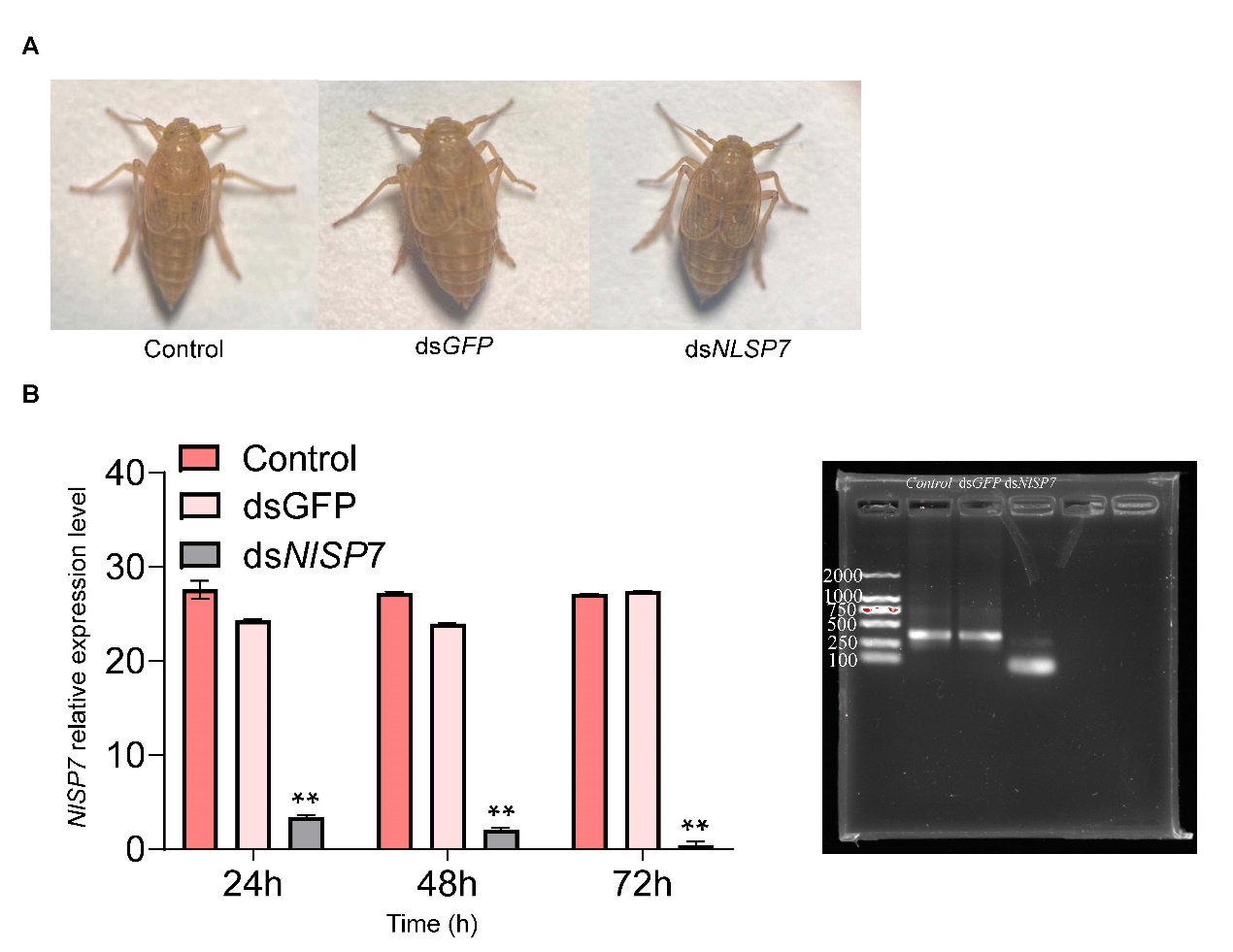


**Supplementary Figure S1.** Brown planthoppers were injected with specific double-stranded RNA. A BPHs after three days of three different treatments, injection of both dsRNAs did not result in any morphological or morphogenetic effect (or phenotype) when compared to uninjected controls. B the expression level of *NLSP7* was determined at 24 h intervals. Data are represented as the mean ± SD from three biological replicates. Asterisks (**) indicate significant difference (P < 0.01, *t*-test). The figure was combined using VHX-7000 digital microscope (KEYENCE, Shanghai, China) and Graphpad Prism version 8.3.1 ([www.graphpad.com](http://www.graphpad.com/)). The gel images of three different treatment groups using GelDoc XR Biorad (Bio-Rad, USA).


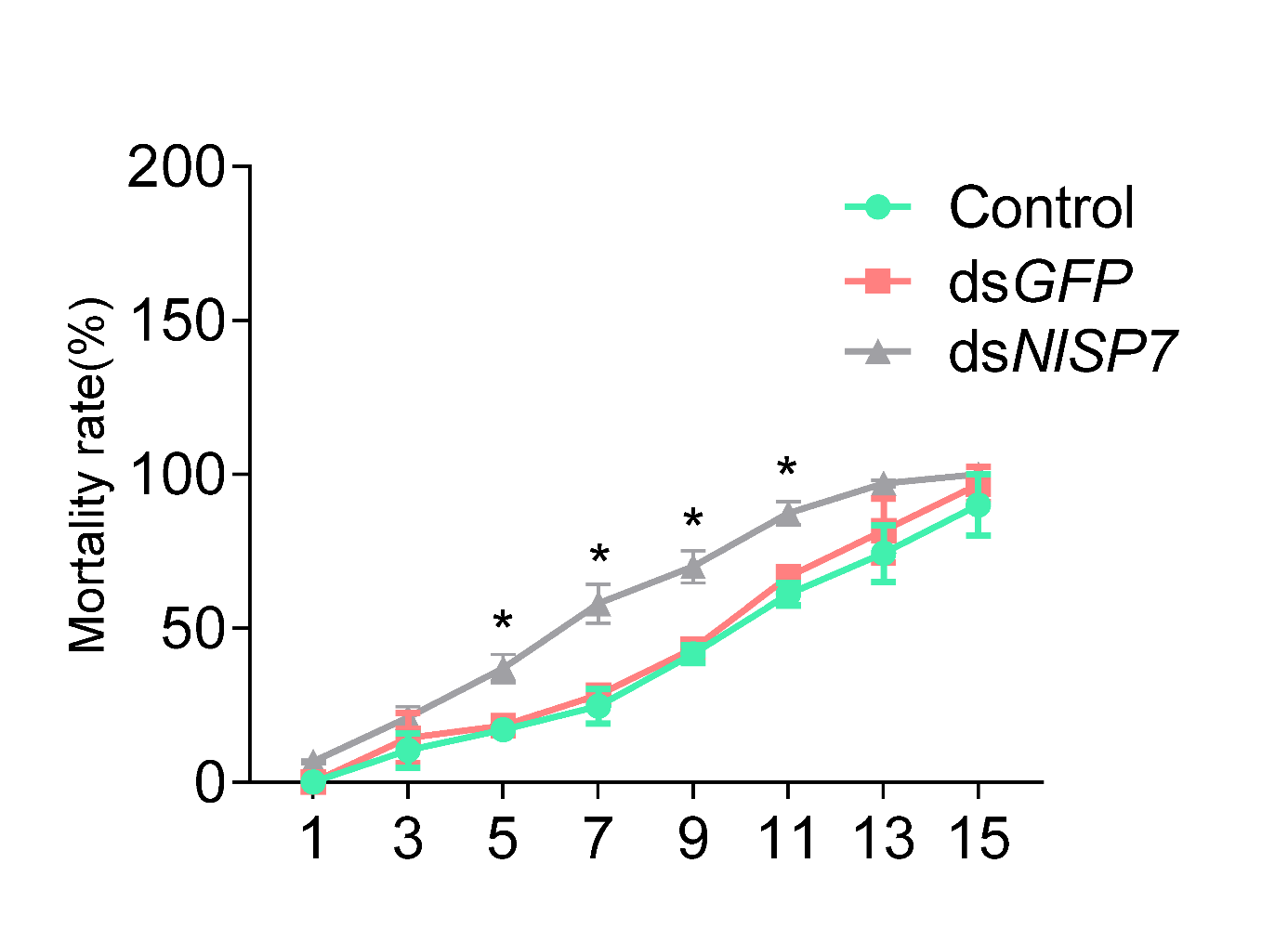


**Supplementary Figure S2.** Mortality rate of BPHs fed TN1 rice, repeated three times, respectively. Each treatment repeated with 100 brown planthoppers. Data are represented as the mean ± SD (*P* < 0.01, Duncan’s multiple range test). The figure was combined using Graphpad Prism version 8.3.1 ([www.graphpad.com](http://www.graphpad.com/)).


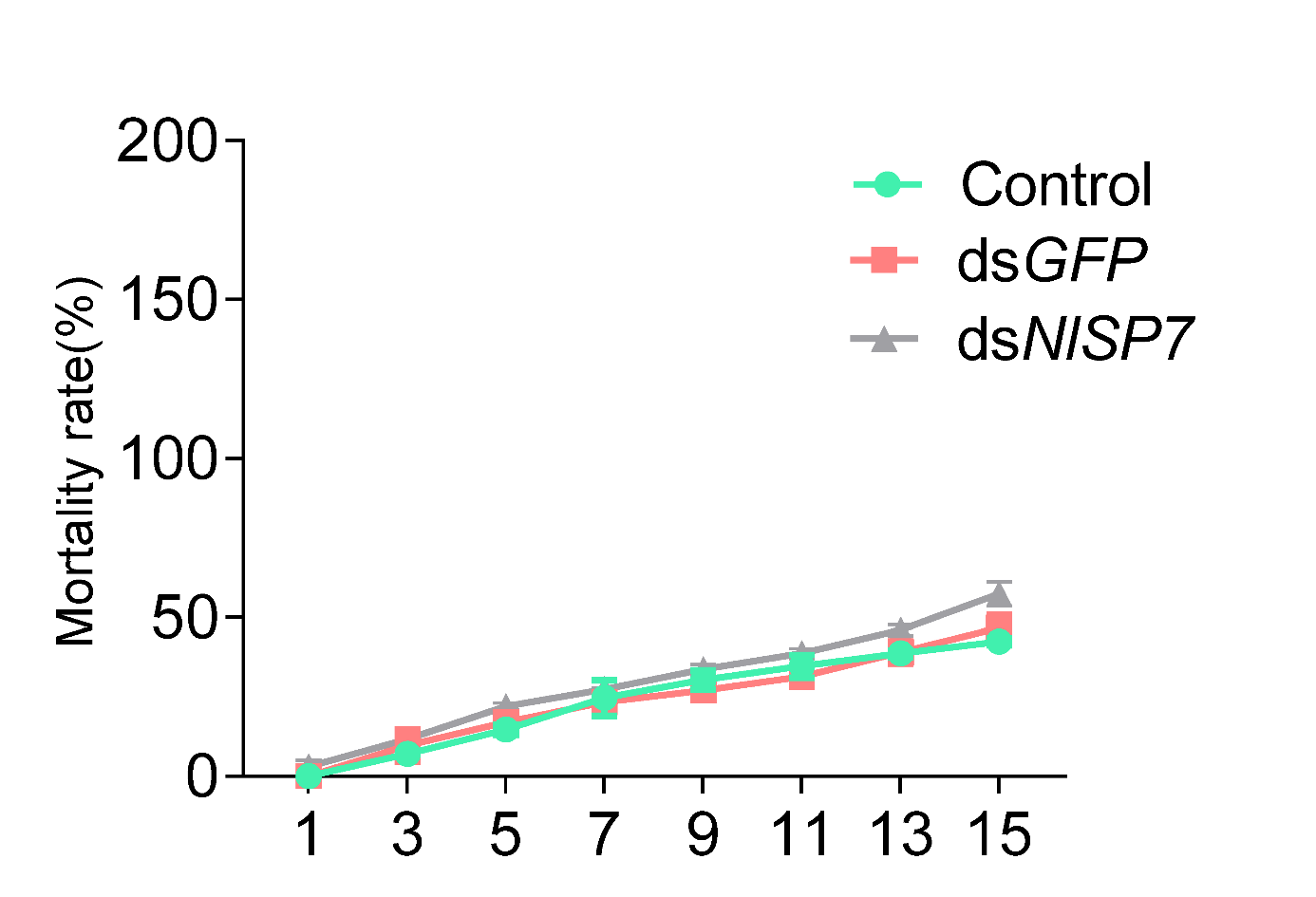


**Supplementary Figure S3.** Mortality rate of BPHs fed an artificial diet with 0 mg/l tricin, no significant difference between three treatments. Each treatment repeated three times. Each treatment repeated with 100 brown planthoppers. Data are represented as the mean ± SD (*P* < 0.01, Duncan’s multiple range test). The figure was combined using Graphpad Prism version 8.3.1 ([www.graphpad.com](http://www.graphpad.com/)).


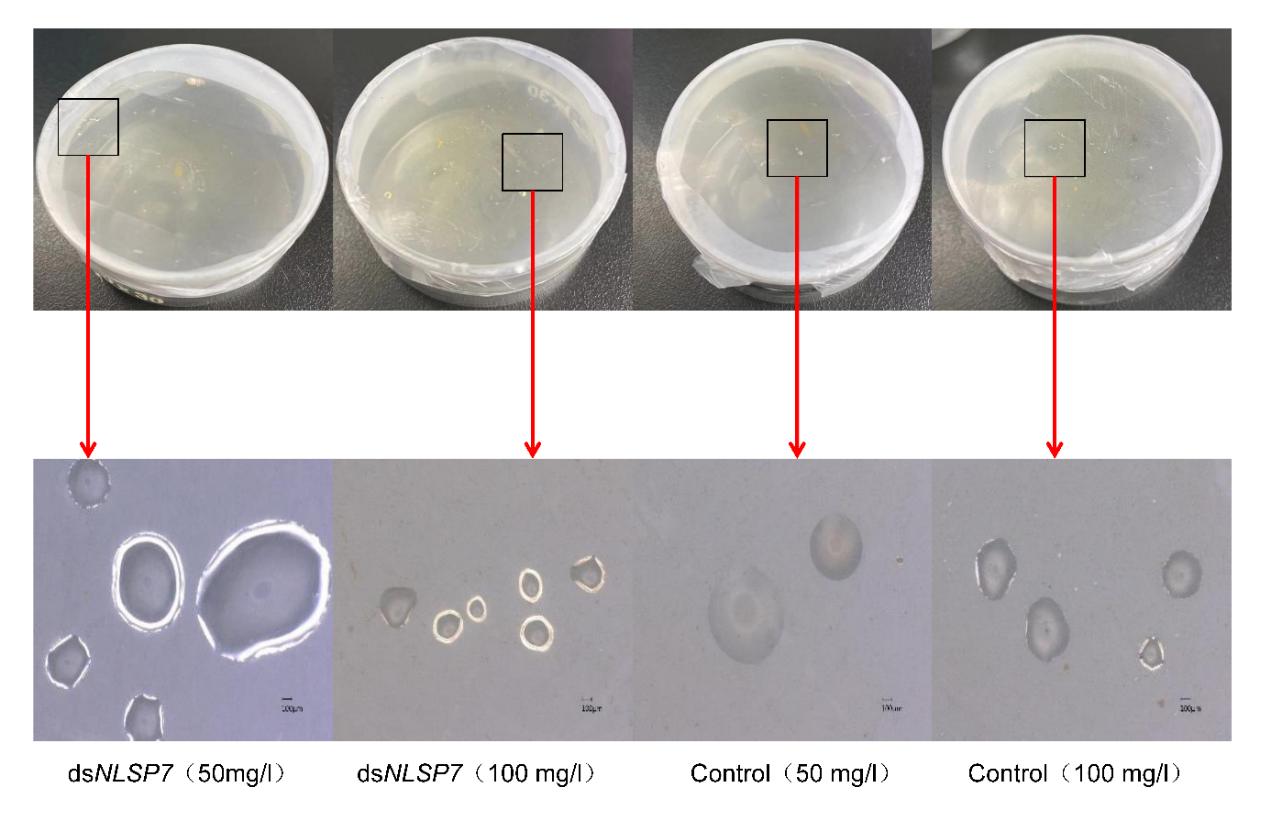


**Supplementary Figure S4.** Effect of *NLSP7* expression silencing on salivary flanges based on an artificial diet with different tricin concentrations. Significant differences were observed for different concentrations of tricin. Each group repeated three times. Different treatment repeated with 100 brown planthoppers. Data are represented as the mean ± SD (*P* < 0.01, Duncan’s multiple range test). The figure was shooted with VHX-7000 digital microscope (KEYENCE, Shanghai, China).

**Supplementary Table S1.** Primers used for qRT-PCR and PCR.

| **Gene** | **Description** | **Forward primer (5'-…-3')** | **Reverse primer (5'…-3')** |  |  |  |  |  |  |  |  |  |  |  |
| --- | --- | --- | --- | --- | --- | --- | --- | --- | --- | --- | --- | --- | --- | --- |
| Nl*actin* | QPCR | ATGAAACCGTCTACAACTCG | GCATCCTGTCGGCAATAC |  |  |  |  |  |  |  |  |  |  |  |
| *NLSP7* | QPCR | AAGAAAGGCAAGAGCAAG | GTAGGCTGCACATAAGGA |  |  |  |  |  |  |  |  |  |  |  |
| *NLSP7* | Cloning | ATGAGGGCTGCCCTGATT | TAGACAACCTGTGGTCCA |  |  |  |  |  |  |  |  |  |  |  |
| *NLSP7* | dsRNA synthesis | GGATCCTAATACGACTCACTATAGG  ATGAGGGCTGCCCTGATT | GGATCCTAATACGACTCACTATAGG  TAGACAACCTGTGGTCCA |  |  |  |  |  |  |  |  |  |  |  |
| *GFP* | dsRNA synthesis | GGATCCTAATACGACTCACTATAGGAA  GGGCGAGGAGCTGTTCACCG | GGATCCTAATACGACTCACTATAGGCA  GCAGGACCATGTGATCGCGC |  |  |  |  |  |  |  |  |  |  |  |
